# Registered nurses’ experiences of community-based nursing in a Swedish context

**DOI:** 10.1186/s12912-025-03636-2

**Published:** 2025-07-23

**Authors:** Snogren Maria, Hagelberg Lena, Lideskar Annelie, Mårtensson Sophie

**Affiliations:** 1 School of Health Sciences, University of Skövde, Högskolevägen, Box 408, Skövde, 541 28 Sweden; 2 Community-Based Nursing, Skövde, Sweden

**Keywords:** Community-based nursing fundamentals of care, Registered nurse

## Abstract

**Background:**

Community-based nursing today faces significant challenges regarding the future supply of healthcare professionals such as registered nurses. This may be due to numerous challenges, such as registered nurses being principally responsible for coordinating care and aligning with the transition to integrated person-centred care, which affects the work environment in community-based nursing. The work environment also affects career advancement prospects, personal motivations, and the desire to make a meaningful contribution to the quality of care. It is, therefore, essential to shed light on registered nurses’ experiences of their working situation within community-based nursing to identify factors that can create a better working situation.

**Aim:**

To describe registered nurses’ experiences of the work situation in Swedish community-based nursing.

**Method:**

The study employed a mixed-methods design. Data were collected through (a) a quantitative sample and (b) a qualitative sample of free-text answers to open-ended questions in the questionnaire. The quantitative material was analysed through descriptive statistics, and the qualitative material was analysed with deductive direct content analysis based on the Fundamental of Care framework’s three dimensions.

**Results:**

The overall experience in community-based nursing was that the registered nurse’s work situation within community-based nursing is influenced by factors such as lack of time and understaffing. Lack of time and understaffing affect the possibilities of creating a caring relationship, the integration of care and the context of care.

**Conclusion:**

Registered nurses’ work situation within community-based nursing is influenced by factors such as lack of time and understaffing. Lack of time and understaffing affect the possibilities of creating a caring relationship with both the person being cared for and their next of kin. In comparison with continuity, good personal knowledge and presence are promoting factors to good caring relationships. The establishment of a care relationship, satisfying basic care and creating organisational conditions take place in collaboration and are the foundations for being able to work person-centred, which also are elements described in the Fundamental of Care framework.

## Background

Sweden’s population is rapidly ageing [1], which requires urgent adaptation and education in community-based nursing [2, 3]. The ageing population contributes to more people with extensive and complex care needs in community-based nursing [4]. Persons with extensive and complex care needs should be able to expect registered nurses with care knowledge and qualified leadership for community-based nursing [5, 6]. The specific knowledge and skills required among registered nurses include knowledge of nursing, medical and public health sciences, ethics, leadership, and pedagogy, as well as a deepened understanding of sustainable development in care and nursing [7]. Registered nurses with knowledge can promote health issues and prevent disease [2, 3] and lead and collaborate with other healthcare professionals [7] which affects the quality of care. The European Specialist Nurses Organization (ESNO) also addresses the evolving roles and the increasing importance of education to bridge policy–practice gaps and improve working conditions [8]. Attitudes toward working with the ageing population [9] and the current shortage of registered nurses and specialist-educated registered nurses in Sweden significantly impact the quality of care in community-based nursing [2, 3]. The quality of care is also affected by the lack of balance between requirements and resources which affects registered nurses’ prioritising in the care [3]. Several government investigations and authorities’ reports also show shortcomings in community-based nursing [10, 11] due to the high workload and mandated overtime among registered nurses [9].

Higher staff turnover in community-based nursing than the labour market’s national average is another challenge in community-based nursing [12]. In 2020 and 2021, registered nurses were the personnel category with the highest turnover in community-based nursing and until 2031, several thousand registered nurses need to be recruited in Swedish community-based nursing, considering the increased number of persons needing healthcare and the number of retirements among registered nurses in the future [4, 12]. The National Board of Health and Welfare’s report also reports that the proportion of specialist registered nurses during the years 2016–2020 decreased by 6% and that region, municipalities and the employment service assess that there is a shortage of specialist registered nurses in the future [4, 12].

The significant challenges regarding the future supply of registered nurses with adequate knowledge and leadership [4, 12] are also affected by high workload and mandated overtime not only leads to health issues for the individual registered nurse but also makes it challenging to recruit new colleagues, which contributes to the global nursing shortage [13]. This may be due to numerous challenges, such as being principally responsible for coordinating care and aligning with the transition to integrated person-centred care [14] which affects the work environment in community-based nursing. The work environment also affects career advancement prospects, personal motivations, and the desire to make a meaningful contribution to the quality of care [15] and is sparsely studied from a Swedish context.

It is, therefore, essential to shed light on registered nurses’ experiences of their working situation within community-based nursing to identify factors that can create a better working situation. Therefore, the study aimed to describe registered nurses’ experiences of their work situation within Swedish community-based nursing.

## Method

The study employed a mixed-methods design, according to Tashakkori and Creswell [16]. Data were collected through (a) a quantitative sample and (b) a qualitative sample of free-text answers from open-ended questions from the questionnaire designed by the author to describe registered nurses’ experiences of their work situation within Swedish community-based nursing. The questions can be seen as being general, overarching mixed-methods questions [16]. The study was evaluated using the Consolidated Criteria for Reporting Qualitative Research (COREQ) checklist to improve the transparency of the research further [17] and the Good Reporting of A Mixed Methods Study (GRAMMS) guidelines [18] to further improve the transparency accuracy in reporting both qualitative and quantitative methods to enable reproducibility of the study.

### Participants and data collection

Participants (*N* = 42) were recruited in the summer and autumn of 2023. The study’s inclusion criteria were registered nurses employed in a medium-sized western Swedish community-based nursing from June to September 2023. Exclusion criteria were temporary workers, parental leave, long-term sick leave and hourly employees. Community-based nursing provides nursing homes, short-term care, nursing homes for people with disabilities and home healthcare. Participants were recruited by providing written and verbal information about the study to the community-based nursing head. The participant characteristics in both parts of the data collection are presented in (Tables 1 and 2).

**Table 1:** Participant characteristics (*N* = 42)

Characteristics	Mean (SD)	Year (min-max)
Age	42 (10)	25–64
Years in profession	14 (10)	1–44
Years in municipality healthcare	8 (7)	1–31
**Workplace**	***n*** **(%)**	
Home healthcare	32 (76)	
Nursing homes ^a)^	10 (24)	

^a)^ Nursing homes including Nursing homes, Short-term care and Nursing homes for people with disabilities

**Table 2:** Description of nurses’ experiences of their work situation within municipal healthcare inspired by the FoC

Question/statement	*n*	Mean (SD)	Very bad/Bad^(a^ *n* (%)	Good/Very good^(b^ *n* (%)
How is the fundamental care provided for patients in municipal healthcare based on Physical needs (hygiene, nutrition, activity, sleep, elimination, physical well-being)?	42	2.95 (0.379)	4 (9)	38 (91)
How is the basic care provided for patients in municipal healthcare based on Psychosocial needs (communication, participation and information, dignity, respect, emotional well-being)?	41	2.90 (0.539)	8 (20)	33 (80)
To what extent do you feel, as a nurse, that you can influence the fundamentals of care?	41	2.59 (0.591)	15 (37)	26 (63)
How are your opportunities as a nurse to establish a good care relationship with your patients based on continuity, participation, reflection and the opportunity for preparation?	40	2.87 (0.607)	10 (25)	30 (75)
How do you feel about the opportunities to work with preventive fundamentals of care?	40	2.40(0.709)	19 (48)	21 (52)
How clear is the mission as a nurse in municipal healthcare to you?	31	2.77 (0.617)	10 (32)	21 (68)
How do you assess your knowledge to carry out your work?	30	3.03 (0.414)	2 (7)	28 (93)
How do you experience access to medical equipment?	31	2.61 (0.615)	12 (39)	19 (61)
How do you feel about the staffing at your unit?	31	2.29 (0.643)	19 (62)	12 (39)
How do you feel about your salary setting in municipal healthcare?	36	2.64 (0.639)	16 (44)	20 (56)
How do you feel that the digital work environment works?	34	2.53 (0.706)	14 (41)	20(59)
How do you experience deviation management in municipal healthcare?	32	2.22 (0.751)	19 (59)	13 (41)
What are your opportunities to influence the decisions that affect the operations at your workplace?	28	2.04 (0.508)	24 (86)	4 (14)

^a)^Very bad/bad 1–2 and ^b)^good/to very good 3–4

### Fundamentals of care

The FoC framework defines FoC as care actions performed by healthcare professionals that respect and focus on a person’s basic needs to ensure their physical and psychosocial well-being, regardless of illness, disease or the environment in which the healthcare is provided [19]. These needs are met by developing a trusting relationship with the person cared for and their next of kin. The care relationship is a dynamic process that includes good knowledge of interpersonal communication among healthcare professionals, moving the care from a series of tasks to coordinated, integrated person-centred care [20–22]. The framework includes three dimensions: [1] Relationship, which includes establishing a relationship; [2] Integration of care, where the basic physical, psychosocial, and relational care needs are identified and met and where a continued care relationship is central; and [3] the context of care, which includes the organisation and policies that include laws, regulations, human resources, and cultural and economic conditions to provide person-centred care. The three dimensions are combined to create a holistic view of person-centred FoC [23].

The authors who are all registered nurses created a questionnaire with both quantitative and qualitative approach questions [16] inspired by the FoC framework’s three dimensions: [1] Relationship [2], Integration of care, and [3] the context of care [23]. The questionnaire was discussed by the authors and pilot-tested among two registered nurses working in a nearby community-based nursing organisation. The final questionnaire included 12 questions with answer options on a Likert scale from very bad to very good on a four-point scale and nine free text questions with the option to answer with free text for the participants to give a deeper perception of the answer. The questionnaire includes subjects such as: To what extent do you feel that you can influence fundamentals of care as a registered nurse? How clear is the assignment as a registered nurse in community-based nursing for you? What are your opportunities to influence the decisions that affect the operations at your workplace? Background questions such as age, workplace, and year of profession were also included in the questionnaire. The COREQ [17] and the GRAMMS guidelines [18] were used by the authors to develop and improve the questionnaire. The questionnaire has not been validated and was developed exclusively for use in this study.

### Data analysis

The quantitative part of the 12 questions in the questionnaire was entered into the International Business Machines Corporation (IBM) Statistical Package for the Social Sciences (SPSS) version 27 [24] for analysis. Descriptive analyses were used to describe differences in the participants’ experience of their work situation within community-based nursing. Descriptive statistics were used to illustrate demographic characteristics.

The text from the nine free-text questions in the questionnaire was analysed with deductive direct content analysis [25] based on the FoC framework’s three dimensions [23]. For the data analysis, first, the text was coded as Hsieh and Shannon [25] described by multiple readings of the answers from the nine free text questions to become familiar with the data and acquire an overview of the texts. Next, the transcripts were carefully reviewed for content. The text corresponding to the three dimensions was highlighted, coded, and transferred into the relevant dimension in the FoC framework. To secure trustworthiness, all authors compared and discussed the text to reach a consensus on the data [25].

### Human ethics and consent to participate

According to the Act 2003:460 [26], no ethical review is required since no personal data has been collected. Yet, relevant parts of the Declaration of Helsinki [27] have been considered and data is stored by the Archive Act [28]. The participants received contact information in case of questions. They were informed that participation was voluntary, that their answers were anonymous and that they consented to participate when filling in the questionnaire.

## Result

The overall perception in community-based nursing was that the registered nurse’s work situation within community-based nursing is influenced by factors such as lack of time and understaffing. Lack of time and understaffing affect the possibilities of creating a caring relationship, the integration of care and the context of care.

### Quantitative part

The majority of the participants rated their opportunities to meet fundamental care needs and the knowledge of their working assignments as ‘good’ or ‘very good’. The overall experience was that they could maintain a ‘good’ or ‘very good’ care relationship. On the other hand, decisions regarding the workplace and the staffing conditions were described as “bad” or very bad. Simultaneously, the participants perceived the salary setting as “good” or “very good”.

Sub-analyses related to participants’ workplace, age, and year of profession showed similar results (data not shown).

### Qualitative part

#### Relationship

Lack of time was perceived by registered nurses as an obstacle to establishing a caring relationship and influenced the opportunity for reflection and preparation. The lack of time meant that prioritisation needed to be done on which persons needed the registered nurses most and resulted in the registered nurses not having the time to meet all the persons for whom they were responsible continuously. One registered nurse perceived it as:

*“There is almost no time to meet the person. There is no time to make the person as involved as one would have liked”.*

Not meeting the persons continuously was a perception among the registered nurses of lacking in healthcare that negatively affected the establishment of the care relationship. Continuity among registered nurses was perceived as a positive way to have a good care relationship. A care relationship can take time and opportunities in community-based nursing to create good care relationships, and then the care extends over a longer period of time. Meeting the person in their home environment was perceived as positive when healthcare problems could be identified by the registered nurses. Essential in the establishment of a caring relationship was described as mutual participation, respect and trust between the registered nurse and the person being cared for and their next of kin.

#### Integration of care

In community-based nursing, the assistant nurse was perceived as performing most of the fundamentals of care and contacting the registered nurse when necessary or when problems arose. The registered nurses were, therefore, dependent on the assistant nurse’s eyes and ears, and their report. The importance of functioning collaboration between registered nurses and other professional categories, such as assistant nurses, was described as a key factor. One registered nurse perceived it as:

The assistant nurse has good insight and together with them, the problems can be identified and addressed in the care.

Continuously holding meetings in which both registered nurses and assistant nurses participated was seen as a functioning collaboration. Preventive work such as preventive measures, and an opportunity to ask questions was given in the meetings. The education level among assistant nurses also affected the fundamentals of care. A High level of education resulted in greater interest and commitment to the person being cared for. A perception among registered nurses was that an increasing number of assistant nurses without caring education, and many of these individuals lack the experience and knowledge to carry out the fundamentals of care. Language barriers were a major problem that created barriers to the care for the person. The missing factors negatively affected care and persons basic care were suffered.

#### Context of care

The work as a registered nurse in community-based nursing was perceived as independent and often involved working alone. The distance between the registered nurses and assistant nurses affected the opportunities for training and supervision by the registered nurses in home healthcare. Registered nurses working in nursing homes had the opportunity and time for daily contact with both assistant nurses and persons they care for, which facilitated training and supervision opportunities. Another registered nurse perceived it as:

*“In community-based nursing*,* good leadership skills are needed when you have to lead the care as a registered nurse”.*

Good teamwork with the assistant nurses was seen when the persons cared for received palliative care (end-of-life care). In palliative care, the registered nurses were more involved in the care, which facilitated training and supervision opportunities. A commitment and knowledge of the organisation were seen but with a desire to influence the structure of the organization. High staff turnover at the management level was highlighted as a factor that affected the organisation’s stability, the possibility of information exchange and long-term investment in the development of care. Suggestions for improvements were maximising the number of assistant nurses per registered nurse, flexible staffing with a registered nurse pool and concrete measures to retain existing registered nurses in the organisation. The digital work environment was perceived as poor and access to on-call support was lacking. The registered nurses perceived the digital equipment as old, and that they worked in many different and incompatible systems.

## Discussion

This study aimed to shed light on registered nurses’ experiences of the work situation within a Swedish community-based nursing organisation. The results highlight some hindering and promoting factors regarding the care relationship. Lack of time and understaffing created obstacles to the care relationship for the registered nurses, resulting in dissatisfaction among both the persons being cared for and their next of kin. Being provided with sufficient time to establish a relationship is described in the results of the present study as a critical factor in the care, and leads to the expansion of professional knowledge, which also is described in relation to the FoC framework. Lack of time for registered nurses resulted in the person being cared for not being involved in the care, which Kitson, Carr [21] also highlight as a key factor in performing good FoC. The results further revealed that the registered nurses had a heavy workload and stressful work environment, hindering their relationship with the person being cared for. Regarding FoC the registered nurse needs to have time and the possibility to have background knowledge and be well-read to create a caring relationship [21]. A prerequisite for understanding the person’s background is security in the education and knowledge of the registered nurse. Furthermore, it is fundamental for the care relationship to be present and to be able to take the time to communicate and confirm what the persons want to convey, aligning with the core essence of nursing practice [21, 29]. The result revealed a difference between registered nurses in nursing homes and in-home healthcare to establish a caring relationship. This may be since at nursing homes; the persons being cared for are in the same place as the registered nurse. The registered nurse in home healthcare rarely meets the persons being cared for daily, and the assistant nurses are in that way their eyes and ears. The result revealed that registered nurses both in home healthcare and in nursing homes experienced that establishing a caring relationship takes time, and that is a different presumption for in-home healthcare and nursing homes.

The results show that registered nurses describe themselves as competent to carry out their work as registered nurses in community-based nursing, but at the same time, highlight a lack of continuous competence development and a need for more registered nurses with specialist training, for example, in the care of the elderly. Specialist training includes knowledge within nursing, medical and public health sciences, ethics, leadership, and pedagogy, as well as a deepened understanding of sustainable development in care and nursing [7]. Fitzpatrick, Hayes [30] previous study shows that specialist training also impacts the care for older adults and will help inform the continuing professional development agenda for registered nurses and allied them to work in this field.

Tiliander, Olsson [15] reveals the importance of the work environment, career advancement prospects, personal motivations, and the desire to make a meaningful contribution to health care in the career choices of registered nurses who attend specialist nursing education. The assistant nurse’s level of competence, experience and language skills are also perceived to be a barrier in the care. By introducing training from both registered nurses and specialist-trained nurses, the conditions could be improved. Registered nurses need support from their managers if they are to be able to get the right conditions for planning their tasks so that professional development becomes a natural part of the work [31, 32].

Leadership or context of care affects employees’ well-being, commitment, and motivation to perform their work. When healthcare professionals enjoy their work, it results in a high standard of quality of care. Through good relations between leaders and employees, trust strongly correlates to employees’ participation and commitment to their work. A leadership imbued with good communication, support and encouragement towards its employees at work leads to less risk of stress and increases motivation [33].

This study shows the registered nurse’s work situation within community-based nursing is influenced by factors such as lack of time and understaffing. Lack of time and understaffing affect the possibilities of creating a caring relationship with both the person being cared for and their next of kin. In comparison with continuity, good personal knowledge and presence are promoting factors to good caring relationships. The establishment of a care relationship, satisfying basic care and creating organisational conditions take place in collaboration and are the foundations for being able to work person-centred which also are elements described in the Fundamental of Care framework.

### Strengths and limitations

This study’s main strength is that it explores a rarely studied area of registered nurses’ experiences of the work situation within Swedish community-based nursing. The results can broaden understanding of the importance of the work environment for registered nurses plays a role in how the provision of fundamentals of care in community-based nursing can be performed. The findings have implications in the practice of healthcare, such as in teaching, research and community-based nursing. Credibility was confirmed by including all authors in the reflective discussions and analysis process. The authors had no close relationship with the informants, which can be seen as a main strength. The study comprised a number of registered nurses with different work experiences to obtain a purposive sample and to ensure dependability, credibility, and transferability [34]. As the questionnaire consisted of both quantitative and qualitative parts, there was an opportunity for the participants to explain their responses and also ensure their understanding of the question. Credibility and reliability were enhanced when all participants answered the same questions. The sample was seen as appropriate when performing this type of study, which strengthens the credibility. However, some limitations must be considered when interpreting the results. One limitation could be that all participants came from the same community-based nursing organisation in an urban area in the western part of Sweden, thereby reducing the transferability of the results to other healthcare areas in need of care. The relatively small sample size could also limit the generalisability of our results, and the questionnaire was explicitly developed for this study.

## Data Availability

The data supporting this study’s findings are available by application to the corresponding author. However, restrictions apply to the availability of these data, which were used under license for the current study and are not publicly available. Data could be made available from the corresponding author on reasonable request.
